# Oleuropein attenuates cardiac fibrosis via modulation of TGF-β1/Smad pathway in diabetic cardiomyopathy rat model

**DOI:** 10.1038/s41598-026-49571-3

**Published:** 2026-05-18

**Authors:** Lobna M. Abdelrauf, Danira A. Habashy, Nadia M. Sharaf, Asmaa A. El-Shafei, Lobna Kassem, Ragwa M. Abdelghany

**Affiliations:** 1 Department of Pharmacology and Toxicology, Faculty of Pharmacy and Biotechnology, German University in Cairo, Cairo, Egypt; 2 Department of Clinical Pharmacy, Faculty of Pharmacy and Biotechnology, German University in Cairo, Cairo, Egypt; 3 Department of Medical Histology and Cell Biology, Faculty of Medicine, Cairo University, Giza, Egypt; 4 Department of Physiology, Faculty of Medicine, Cairo University, Giza, Egypt

**Keywords:** Oleuropein, Cardiac fibrosis, Diabetic cardiomyopathy, TGF-β1/Smad signaling, Cardiology, Cell biology, Diseases, Medical research, Molecular biology

## Abstract

Cardiac fibrosis is a key pathological feature of diabetic cardiomyopathy (DCM), contributing to progressive cardiac dysfunction. Transforming growth factor beta-1 (TGF-β1), via Smad2/3 signaling pathway, promotes fibrotic gene expression. Oleuropein (OL), a major phenolic constituent of olive products, exhibits significant antioxidant properties and contributes to cardiovascular protection. This study aimed to investigate the anti-fibrotic effects of OL in a rat model of DCM and its modulation of the TGF-β1/Smad signaling pathway, using Losartan as a positive control. Thirty-two male Wistar rats were randomly assigned to four groups: control, DCM, DCM + OL (40 mg/kg), and DCM + Losartan (10 mg/kg). DCM was induced via high-fat diet (HFD) and a single low-dose streptozotocin (STZ) injection (40 mg/kg). Cardiac function was assessed by echocardiography. Histological evaluation included hematoxylin–eosin (H&E) and Masson trichrome staining. Cardiac biomarkers, oxidative stress markers, and fibrosis-related markers were analyzed using RT-qPCR, immunohistochemistry, and western blotting. DCM rats exhibited cardiac dysfunction and fibrosis. OL treatment significantly improved cardiac function, reduced collagen and fibronectin (FN) deposition, and downregulated TGF-β1, Smad2/3, α-smooth muscle actin (α-SMA), collagen types I/III, matrix metalloproteinases-2/9 (MMPs), and tissue inhibitor of metalloproteinases-1 (TIMP-1), while enhancing Smad7 expression. Besides, OL demonstrated notable antioxidant and antidiabetic effects. In conclusion, OL alleviates cardiac fibrosis and dysfunction in DCM, potentially through modulation of the TGF-β1/Smad signaling, highlighting its therapeutic promise in diabetic cardiac complications.

**Supplementary Information:**

The online version contains supplementary material available at 10.1038/s41598-026-49571-3.

## Introduction

Diabetic cardiomyopathy, an independent cardiovascular complication of diabetes mellitus, is characterized by structural and functional myocardial remodeling, primarily driven by cardiac fibrosis^1^. Oxidative stress, inflammation, and excessive extracellular matrix (ECM) deposition, particularly collagens, disrupt ventricular mechanics, resulting in impaired compliance and progressive dysfunction^2,3^. Central to this fibrotic process is TGF-β1 signaling, which promotes fibroblast activation and differentiation into α-SMA positive myofibroblasts^4^. Myofibroblasts are the key cellular effectors of cardiac fibrosis, inducing the synthesis of collagens type I and III and FN, while secreting MMPs and their inhibitors, TIMPs, thereby regulating ECM turnover^5,6^. MMPs, particularly MMP-2 and MMP-9, also play roles beyond ECM degradation, including modulation of inflammatory responses and activation of latent TGF-β1, thereby further accentuating the fibrotic process^7^. TGF-β1 mediates cardiac fibrosis via Smad2/3-Smad4 signaling, which activates profibrotic gene transcription, while Smad6/7 act as negative regulators, with Smad7 additionally promoting receptor degradation^8,9^.

Dietary interventions are increasingly recognized for their potential to alleviate diabetic complications. Olive oil is rich in biophenols such as OL which possesses well-documented antioxidant, anti-inflammatory, antidiabetic, and cardioprotective properties^10,11^. Although other naturally derived plant compounds, such as resveratrol and curcumin, have shown anti-fibrotic promise in various models of cardiomyopathy^12,13^, OL represents a distinctly relevant alternative given its central role in the cardioprotective Mediterranean diet and its potent capacity to simultaneously neutralize oxidative stress and chronic inflammation; the primary upstream triggers of TGF-β1 activation in the diabetic heart. Nevertheless, a critical knowledge gap persists: despite extensive characterization of OL’s broader biological activities, its direct regulatory impact on the pro-fibrotic TGF-β1/Smad axis remains poorly defined.

The present study investigates the anti-fibrotic efficacy of OL as a therapeutic intervention in a rat model of DCM induced by HFD and STZ. The impact of OL on cardiac structure and function is evaluated, relative to Losartan, a recognized anti-fibrotic agent. Additionally, we explore the involvement of the TGF-β1/Smad signaling pathway as a potential mechanism underlying the therapeutic effects of OL. Through this investigation, we seek to elucidate the role of OL in attenuating cardiac fibrosis, thereby supporting its development as a promising dietary-based intervention for DCM.

Most experimental designs evaluate preventive strategies; however, to maximize clinical relevance, the present study employs a therapeutic strategy, initiating intervention only after the onset of stable hyperglycemia and early diabetic complications.

## Methods and materials

### Experimental protocol

Thirty-two male Wistar rats (weight: 200-250g) were acquired from the National Organization for Drug Control and Research (NODCAR) animal house in Egypt. Rats were housed in transparent plastic cages (4 rats/cage) under controlled environmental conditions (22–24 °C temperature, 50–55% humidity, and 12-h light/dark cycle). The experimental protocol was in accordance with the guidelines for the care and use of laboratory animals established by the US National Institute of Health (NIH publication 85–23 revised 1996). Study procedures were approved by the Ethics Committee of the German University in Cairo, Egypt (ID no. PTX-2019–05-LAK-1) and adhered to the ARRIVE 2.0 guideline.

Rats were randomly divided into four groups (n = 8 rats/group); Group I (Control), Group II (DCM), Group III (DCM + OL), and Group IV (DCM + Losartan). To minimize bias the investigators conducting experimental analyses were blinded to the animal group assignments. The sample size was calculated according to ARRIVE 2.0 guideline^14^, utilizing an α-level of 0.05 and a statistical power = 0.8. All rats had free access to water and food. Control rats received standard rat chow (3 kcal/g) providing 12% kcal as fat, while diabetic rats, Groups II, III and IV, received HFD (4.62 kcal/g; in-house prepared sheep-tail fat mixed with standard chow) providing 60% kcal as fat^15,16^ (Table S1). Rats were maintained on their respective diet during the whole experimental period, and their body weights (BW) were recorded weekly.

To induce a diabetic state reflecting key metabolic features of type 2 diabetes milieus (T2DM), rats received HFD feeding for 2 weeks followed by a single intraperitoneal (*i.p.*) STZ injection (40 mg/kg, dissolved in 0.1M sodium citrate buffer, pH 4.5 at 4°C)^17^. Control animals received an equivalent volume of citrate buffer alone. Fasting blood glucose levels were measured after an overnight fast using a glucometer (Accu-Chek Go model GS; Roche, Germany) via tail vein, one week following STZ injection. Inclusion criteria were fasting blood sugar ≥ 250 mg/dL^18^.

Following the initial 2-week dietary induction period, animals were maintained for an additional 6 weeks after the STZ injection. This timeline has been established in recent literature to reliably induce early functional and structural manifestations of DCM, including cardiac hypertrophy and fibrosis^19,20^. Afterwards, treatment commenced in diabetic Groups III and IV. Group III (DCM + OL group) diabetic rats were administered OL (Carbosynth Limited, Berkshire, UK) (40 mg/kg) *i.p.* daily for a further 6 weeks, while Group IV (DCM + Losartan group) rats were administered Losartan (Sigma, St. Louis, USA) (10 mg/kg) *i.p.* daily for a further 6 weeks, serving as a positive control given its recognized anti-fibrotic efficacy. Group II (DCM group) diabetic rats, however, continued on their HFD without receiving any treatment. Both OL and Losartan were dissolved in sterile distilled water.

It should be noted that the scope of the current study was to assess a single dosage regimen for OL, selected based on previous studies demonstrating optimal efficacy in fibrotic models without inducing acute distress^21–23^ rather than evaluating multiple doses. Additionally, the assessment of systemic toxicity (hepatic and renal markers) and the measurement of plasma pharmacokinetic parameters to confirm cardiac bioavailability of OL were outside the scope of the current experimental design.

### Echocardiography

At the end of the experimental period, transthoracic echocardiography was performed to evaluate cardiac function using an Esaote MyLab™ 30Gold Vet ultrasound system (Genova, Italy) equipped with an 8-MHz transducer, as previously described^24^. Echocardiography was evaluated solely at the endpoint of the study. However, because treatment was initiated only after the disease model was established, the magnitude of structural and functional remodeling was accurately assessed by comparing all endpoint echocardiographic parameters from the diabetic cohorts against an age-matched, healthy negative control group. Rats were anesthetized with *i.p.* sodium pentobarbital (100 mg/kg), the thoracic region was shaved, and animals were placed in the supine position. Ultrasound gel was applied to the thorax to improve visibility. Two-dimensional guided M-mode images were obtained from the left parasternal short-axis view of the LV at the level of the papillary muscles. Left ventricular diameters at end-diastole (LVDd) and end-systole (LVDs) were measured from the M-mode tracings, and ejection fraction (EF%) was recorded as an index of LV systolic function.

### Sample preparation

At the end of the study, rats were anesthetized with *i.p.* sodium pentobarbital (100 mg/kg) after 8 h fasting^25^. Blood samples were collected from the retro-orbital venous plexus using heparinized capillary tubes, centrifuged at 3,000 rpm for 15 min and stored in – 80 °C for biochemical analysis. Anesthetized rats were then sacrificed by cervical dislocation and hearts were excised, weighed, and dissected. Left ventricular (LV) strips were fixed in 10% buffered formalin for histological and immunohistochemical evaluation. The remaining of LVs were stored at -80 °C for gene and protein analysis.

### Serum biochemical analysis

Fasting serum glucose level was analyzed by colorimetric kit (Diamond Diagnostics Kits, Cairo, Egypt) as described by *Trinder *^26^. Fasting serum insulin was determined using rat insulin ELISA kit (Cohesion Biosciences, London, UK) according to the manufacturer’s protocol. Insulin resistance index (HOMA-IR) was then calculated according to the following formula: HOMA-IR = [Fasting insulin (μIU/ml) x Fasting glucose (mmol/L)] / 22.5^27^.

Creatine Kinase-MB (CK-MB) activity was determined in serum using BioMed Diagnostics CK-MB Kit (Hannover, Germany) and was used along with the heart weight (HW) to BW ratio to assess heart structure. Lipid peroxidation was estimated by measuring serum malondialdehyde (MDA) and serum superoxide dismutase (SOD) activity via colorimetric assays (Bio-Diagnostics, Giza, Egypt).

### Quantitative real-time RT-PCR analysis

Expression levels of B-type Natriuretic peptide (BNP), collagen I, collagen III, α-SMA, MMP-2, MMP-9, TIMP-1, TGF-β1, Smad2, Smad3 and Smad7 genes were determined using RT-qPCR technique. Total RNA was extracted from LV tissue using RNeasy Mini kit (Qiagen, Hilden, Germany) according to manufacturer’s instructions and then was reversibly transcribed into single-stranded cDNA using High-Capacity cDNA Reverse Transcription Kit (Applied Biosystems, Vilnius, Lithuania). qPCR was performed using TaqMan gene expression assay using TaqMan FAM/MGB probes, endogenous control Rat β-actin (ACTB) VIC/MGB probe (Applied Biosystems, Vilnius, Lithuania) and TaqMan Master mix (Applied Biosystems, Vilnius, Lithuania).

Gene expression analysis was performed using Applied Biosystems StepOnePlus™ Real-Time PCR System according to the manufacturer’s protocol. Thermal cycling conditions consisted of an initial step at 50 °C for 2 min and 95 °C for 10 min, followed by 40 cycles of 95 °C for 15 s and 60 °C for 1 min.

The TaqMan gene expression assays used were as follows; ACTB (Rn00667869_m1), NPPB (Rn00580641_m1), ACTA2 (Rn01759928_g1), COL1A1 (Rn01463848_m1), COL3A1 (Rn01437681_m1), MMP2 (Rn01538170_m1), MMP9 (Rn00579162_m1), TIMP1 (Rn01430873_g1), TGFB1 (Rn00572010_m1), SMAD2 (Rn00569900_m1), SMAD3 (Rn00565331_m1), and SMAD7 (Rn01523958_m1).

For normalization of target gene expression, β‑actin was selected as the reference gene. To ensure its suitability, we validated β‑actin expression stability across all experimental groups (control, diabetic, and treatment). Cycle threshold (Ct) values were analyzed for consistency, and no significant variation was observed between groups. The mRNA levels of target genes were then normalized to their respective β-actin and compared to the control. The relative gene expression was calculated using the ΔΔCt method (fold change)^28^.

### Western blot analysis

Protein expression levels of TGF-β1 and phosphorylated Smad3 (*p*-Smad3) were evaluated by western blot analysis to complement the mRNA expression findings. LV tissues were snap-frozen in liquid nitrogen and homogenized using a rotor–stator homogenizer in ice-cold IP/Western Lysis Buffer supplemented with protease and phosphatase inhibitors (Bio Basic Inc., Ontario, Canada). Total protein concentrations were quantified using the Bradford Protein Assay Kit (Bio Basic Inc., Ontario, Canada), following the manufacturer’s protocol. Protein samples (20 µg) were mixed 1:1 with 2 × Laemmli Sample Buffer (Bio-Rad, CA, USA) and denatured at 95 °C for 5 min.

Proteins were separated by 10% SDS–polyacrylamide gel electrophoresis (SDS-PAGE) at 150 V for 1 h and transferred using a semi-dry transfer system onto polyvinylidene fluoride (PVDF) membranes (Bio-Rad, CA, USA) pre-activated in 100% methanol and equilibrated in 1 × Tris–Glycine Transfer Buffer. To optimize antibody hybridization, membranes were horizontally cut into strips based on molecular weight markers. Following transfer, membranes were blocked for 1 h at room temperature in 5% bovine serum albumin (BSA) in Tris-buffered saline containing 0.1% Tween-20 (TBST).

Membranes were then incubated overnight at 4 °C with primary antibodies: anti-TGF-β1 (1:1000; Abcam, ab92486), anti-phospho-SMAD3 (Ser423/425) (1:1000; Abcam, ab52903), and anti-glyceraldehyde 3-phosphate dehydrogenase (GAPDH) (1:5000; Abcam, ab181602) used as internal reference. While evaluating the ratio of phosphorylated to total Smad proteins is standard practice, total Smad3 protein levels were not assessed in this specific blot cohort; thus, *p*-Smad3 was normalized directly to GAPDH to reflect absolute levels of the activated protein.

Following washing with TBST, membranes were incubated for 1 h at room temperature with HRP-conjugated goat anti-rabbit IgG secondary antibody (1:5000; Abcam, ab6721). Blots were visualized using enhanced chemiluminescence (ECL) and quantified using ImageJ (NIH) image analysis software. Target protein expression levels were normalized to GAPDH, and relative intensities were compared to the control^29^.

### Histopathological examination

Heart tissue samples fixed in 10% formalin and embedded in paraffin were cut into 5-μm-thick sections for histopathological examination; including *H&E staining* for tissue morphological assessment, and *Masson’s Trichrome staining* for the detection of collagenous matrix. The resulting fibrotic area was quantified using computer-assisted image analysis. In addition, *Immunohistochemical staining* was made for FN and α-SMA protein expression analysis.

Cardiac tissue sections were de-paraffinized, rehydrated through graded alcohols, and then pretreated with 0.1 M citrate buffer (pH 6.0), 2 times for 5 min, in a microwave for antigen retrieval. Sections were incubated overnight at 4 °C with one of the following primary antibodies: rabbit polyclonal anti-FN (Abcam, ab2413, Cambridge, UK) and rabbit monoclonal anti- α-SMA (Abcam, ab124964, Cambridge, UK) diluted 1:300 in phosphate-buffered saline (PBS). The following day, sections were incubated at room temperature for 2 h with HRP-conjugated goat anti-rabbit IgG (Abcam, ab205718, Cambridge, UK) diluted 1:2000 in PBS. After that, sections were incubated with 3, 3′-Diaminobenzidine (DAB) (Sigma, St. Louis, USA) as chromogen for 5 min followed by counterstaining with Mayer’s hematoxylin. Stained sections were examined under a light microscope (Leica, Germany), and images were captured at 400 × magnification. Quantitative analysis of positive staining was performed using Image-Pro Plus 6.0 software. For each tissue sample, ten non-overlapping fields were randomly selected using systematic sampling to determine the optical density of FN and α-SMA staining. The ratio of FN-positive and α-SMA-positive areas to total heart area was calculated to quantify protein accumulation^30^.

### Statistical analysis

Statistical analysis was performed using GraphPad Prism version 8 (GraphPad Software Inc., San Diego, CA, USA). Samples were randomly assigned to their respective experimental groups, with a sample size of n = 8 per group. Data are expressed as mean ± standard error of the mean (SEM). The normality of the distribution was verified using the Shapiro–Wilk test, and homogeneity of variance was confirmed using the Brown-Forsythe test. For comparisons between multiple groups, one-way analysis of variance (ANOVA) was employed, followed by Tukey’s test for inter-group comparisons. Results were considered statistically significant when the *p*-value was less than 0.05. For primary functional and structural outcomes, the magnitude of the biological effect is reported in the text as the absolute mean difference alongside the 95% confidence interval (95% CI). The mean differences and 95% CIs for all remaining systemic, gene, and protein expression comparisons are detailed in the Supplementary Table S2.

## Results

### Metabolic characterization

Metabolic characterization was assessed based on alteration in rats BW, fasting serum glucose levels, circulating insulin concentrations, and the HOMA-IR index. Administration of STZ combined with HFD feeding successfully induced a diabetic phenotype reflecting key metabolic features of T2DM, characterized by insulin resistance with partial β-cell dysfunction.

Compared to the control group, diabetic rats exhibited significant hyperglycemia (Mean Difference =  + 203.5 mg/dL, 95% CI [175.6 to 231.4], *p* < 0.001), reduced circulating insulin concentration, and a marked increase in the HOMA-IR index, indicating the presence of insulin resistance accompanied by partial β-cell dysfunction and impaired insulin secretion.

Treatment with OL and Losartan significantly reduced fasting serum glucose levels compared to the untreated DCM group (OL vs. DCM: Mean Difference = − 163.3 mg/dL, 95% CI [− 191.2 to − 135.4], *p* < 0.001; Losartan vs. DCM: Mean Difference = − 152.3 mg/dL, 95% CI [− 180.2 to − 124.4], *p* < 0.001), partially restored circulating insulin concentrations, and attenuated the diabetes-induced increase in HOMA-IR. These findings confirm successful establishment of the experimental model and indicate that both treatments improved systemic metabolic control (Table 1). Because OL and Losartan both significantly improved blood glucose profiles, we cannot definitively rule out systemic glycemic reduction as a contributing mechanism of cardioprotection. However, as detailed in subsequent sections, the pronounced modulation of specific fibrotic pathways suggests direct myocardial benefits that extend beyond glycemic control alone.

**Table 1:** Metabolic characterization, body weight and HW/BW ratio.

	Control	DCM	DCM + OL	DCM + Losartan
Serum glucose (mg/dl)	91.21 ± 4.78	294.7 ± 9.13 *	131.4 ± 6.59 ^#^	142.6 ± 7.31 ^#^
Serum insulin (ng/ml)	1.62 ± 0.14	1.02 ± 0.04 *	1.41 ± 0.12 ^#^	1.37 ± 0.11
HOMA-IR	8.98 ± 0.55	18.26 ± 0.49 *	11.31 ± 0.93 ^#^	11.89 ± 0.86 ^#^
HW (mg)	965.75 ± 122.2	1143.96 ± 189.5	1002.91 ± 205.3	1045.65 ± 172.9
BW (g) (at end of study)	327.37 ± 16.1	216.25 ± 14.7 *	231.62 ± 17.2	239.28 ± 18.6
HW/BW (mg/g)	2.95 ± 0.14	5.29 ± 0.12 *	4.33 ± 0.18 ^#^	4.37 ± 0.15 ^#^

Values are expressed as mean ± SEM (n = 8). *p < 0.05 vs. control group; ^#^*p* < 0.05 vs. DCM group.

HOMA-IR: homeostatic model assessment of insulin resistance; BW: body weight; HW: heart weight.

### BW changes and HW/BW ratio

Control rats showed a progressive increase in BW during the experimental period. Whereas diabetic rats exhibited a significant reduction in final BW following STZ injection, despite prior exposure to HFD. This reduction is consistent with the metabolic consequences of insulin deficiency and resistance, which promote enhanced proteolysis and lipolysis. Treatment with OL and Losartan resulted in a modest increase in final BW compared to untreated diabetic rats, although the difference did not reach statistical significance (Fig. S1, Table 1).

The heart weight-to-body weight ratio (HW/BW) was significantly elevated in the DCM group compared to control animals, indicating diabetes-associated cardiac remodeling. Both OL and Losartan treatments significantly reduced the HW/BW ratio compared to untreated diabetic rats (Table 1).

### Echocardiography

Echocardiographic evaluation revealed significant structural and functional alterations in diabetic rats. While baseline echocardiography prior to treatment initiation was not performed to confirm pre-existing early remodeling, endpoint evaluation of the DCM group exhibited marked LV dilation, evidenced by significant increases in LVDd and LVDs, accompanied by a significant reduction in EF% compared to the control group (Mean Difference = − 20.25%, 95% CI [− 32.89 to − 7.61], *p* < 0.001), indicating impaired systolic function. These findings confirm the progressive development of diabetes-induced cardiac dysfunction.

**Fig. 1:**
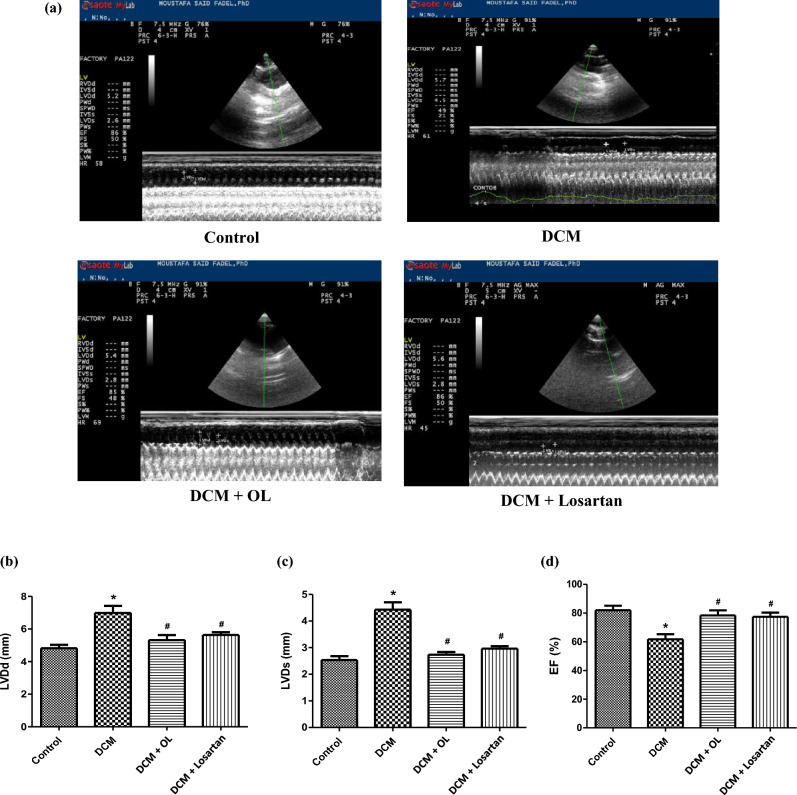
Echocardiographic assessment of cardiac function. **(a)** Representative M-mode echocardiographic images obtained from the experimental groups at the end of the study period. Quantitative analysis of **(b)** LVDd, **(c)** LVDs, and **(d)** EF%. Results are expressed as mean ± SEM (n = 8). *p < 0.05 vs. control group; ^#^*p* < 0.05 vs. DCM group. LVDd: left ventricular diameter at end-diastole; LVDs: left ventricular diameter at end-systole; EF: ejection fraction. Experimental groups: Control (normal diet), DCM (diabetic cardiomyopathy induced by HFD + low-dose STZ), DCM + OL (oleuropein-treated diabetic rats), and DCM + Losartan (losartan-treated diabetic rats).

Treatment with OL and Losartan significantly attenuated these abnormalities. Both treatments reduced LVDd and LVDs relative to untreated diabetic rats and significantly improved EF%, indicating partial restoration of cardiac contractile performance (OL vs. DCM: Mean Difference =  + 16.63%, 95% CI [3.98 to 29.27], *p* < 0.01; Losartan vs. DCM: Mean Difference =  + 15.63%, 95% CI [2.98 to 28.27], *p* < 0.05). Overall, these results demonstrate that OL and Losartan preserve cardiac function and alleviate diabetes-induced ventricular remodeling (Fig. 1a–d).

### Cardiac histology and collagenous matrix deposition

Histological examination of myocardial tissue using H&E staining revealed well-organized cardiac muscle fibers with preserved architecture in the control group. In contrast, cardiac tissue from DCM rats exhibited pronounced structural abnormalities characterized by disorganization of myocardial fibers, congestion and dilation of blood vessels, and infiltration of fibroblasts, indicating extensive myocardial injury and structural remodeling.

**Fig. 2:**
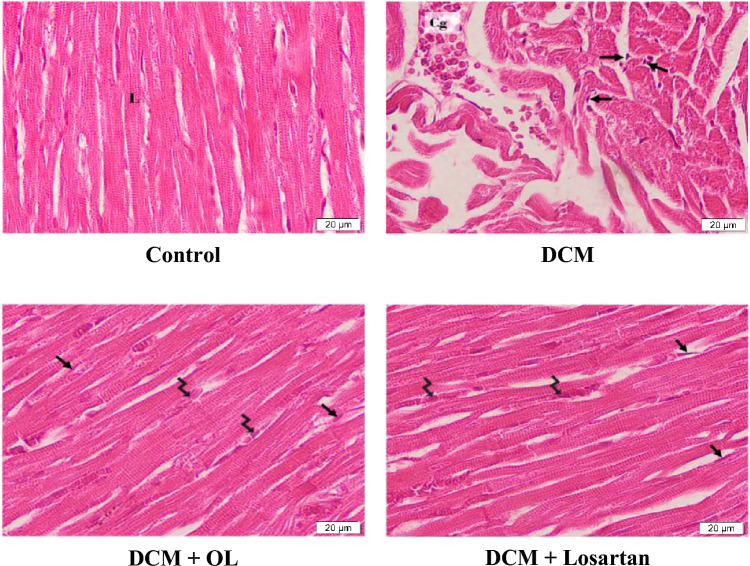
Histological assessment of myocardial architecture by H&E staining. **(a)** Representative Hematoxylin and Eosin (H&E) staining of cardiac tissue showing myocardial structural alterations among experimental groups (magnification 400 × , scale bar 20 μm). [L] indicates longitudinal arrangement of cardiac muscle fibers; [Cg] indicates congestion and dilatation of blood vessels; [Arrow] indicates fibroblasts infiltration; [Zig zag arrow] indicates restoration of longitudinal myocardial fiber organization in treated groups.

Administration of OL and Losartan markedly improved myocardial architecture. Cardiac sections from treated groups displayed partial restoration of the longitudinal arrangement of cardiac muscle fibers with reduced fibroblast infiltration, suggesting attenuation of diabetes-induced myocardial damage (Fig. 2).

**Fig. 3:**
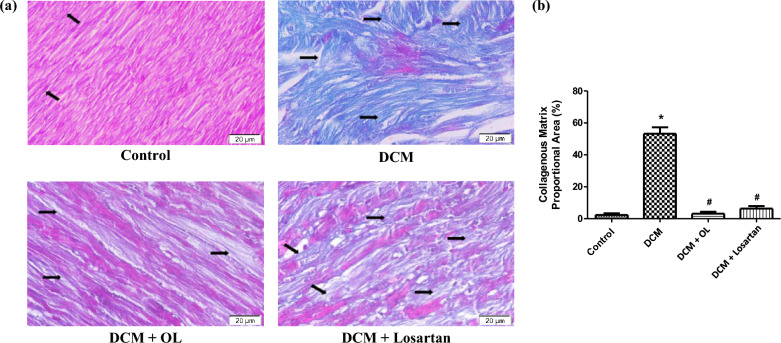
Evaluation of cardiac collagen deposition by Masson’s trichrome staining. (**a**) Representative Masson’s trichrome staining of cardiac tissue showing collagenous matrix deposition (magnification 400 × , scale bar 20 μm). Collagen fibers are stained blue [Arrow]. (**b**) Quantitative analysis of collagen deposition expressed as percentage area of collagen-positive staining. Values are expressed as mean ± SEM. *p < 0.05 vs. control group; ^#^*p* < 0.05 vs. DCM group.

Masson’s trichrome staining further demonstrated significant ECM collagen accumulation in the myocardium of DCM rats compared to control animals. Quantitative analysis, measured as the percentage of fibrotic area relative to total tissue area, confirmed a significant increase in collagen deposition in the DCM group (Mean Difference =  + 50.87%, 95% CI [37.85 to 63.89], *p* < 0.001). Statistical comparisons across all experimental groups demonstrated that treatment with OL and Losartan significantly reduced collagen accumulation relative to untreated diabetic rats (OL vs. DCM: Mean Difference = -50.05%, 95% CI [– 63.07 to – 37.03], *p* < 0.001; Losartan vs. DCM: Mean Difference = -46.84%, 95% CI [− 59.86 to − 33.82], *p* < 0.001), indicating robust attenuation of myocardial fibrosis and ECM remodeling (Fig. 3a, b).

### Immunohistochemical staining

Immunohistochemical analysis revealed marked ECM remodeling in diabetic myocardium. Cardiac tissue from the DCM group demonstrated a substantial increase in FN and α-SMA immunoreactivity compared to the control group, indicating enhanced fibroblast activation and myofibroblast differentiation.

**Fig. 4:**
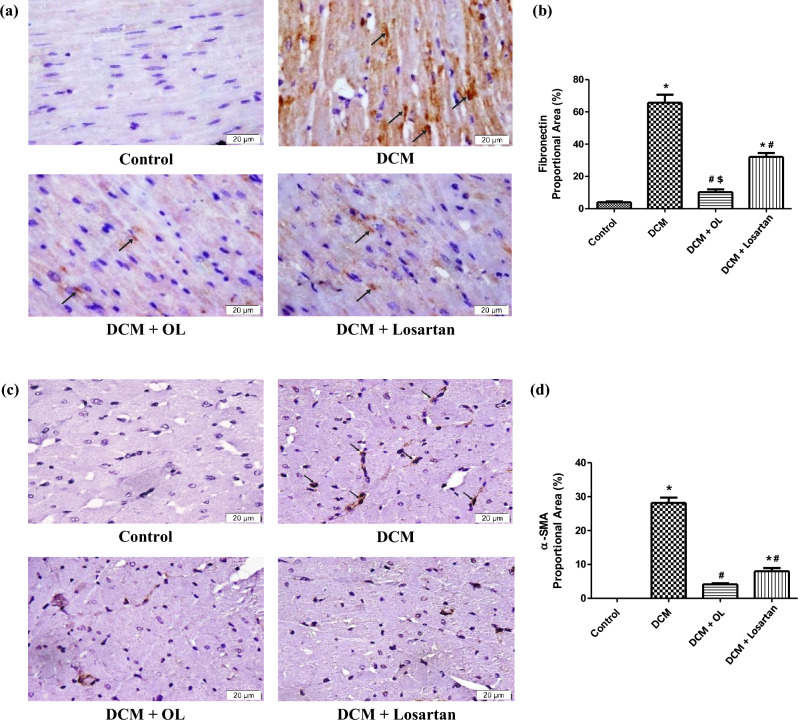
Immunohistochemical detection of fibronectin and α-SMA in cardiac tissue. Representative immunohistochemical staining for **(a)** FN and **(c)** α-SMA in cardiac tissue sections (Magnification 400 ×). Positive staining appears brown [Arrow]. Quantitative analysis of the percentage area of positive staining is shown for **(b)** FN and **(d)** α-SMA. Values are expressed as mean ± SEM. *p < 0.05 vs. control group; ^#^*p* < 0.05 vs. DCM group; ^$^*p* < 0.05 vs. Losartan-treated group. FN: fibronectin; α-SMA: alpha-smooth muscle actin.

Treatment with OL and Losartan significantly attenuated FN and α-SMA deposition in cardiac tissue. Quantitative analysis of the positively stained areas confirmed a significant reduction in both markers in the treated groups relative to the DCM model. Notably, OL treatment produced a more pronounced reduction in FN deposition compared with Losartan. These findings indicate that OL and Losartan suppress fibroblast activation and ECM remodeling in diabetic myocardium (Fig. 4a–d).

### Cardiac biomarkers and oxidative stress

Assessment of cardiac injury markers revealed significant myocardial damage in diabetic rats. The DCM group exhibited a marked increase in BNP gene expression in cardiac tissue together with elevated serum CK-MB activity compared to the control group, indicating cardiac myocyte stretching and injury.

**Fig. 5:**
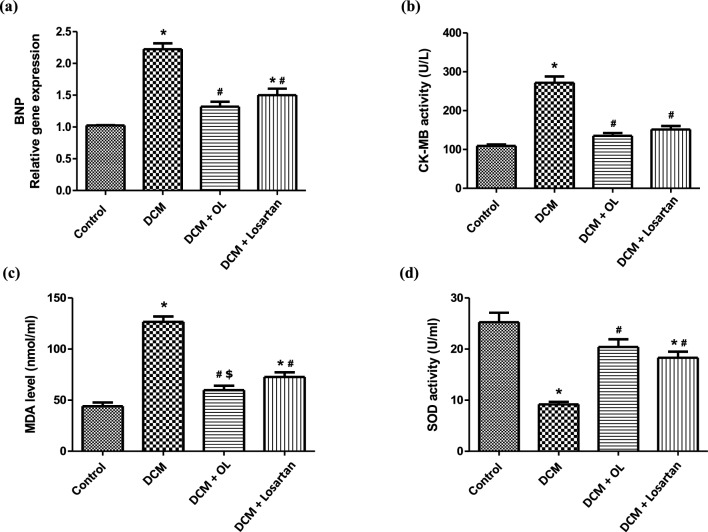
Cardiac injury and oxidative stress biomarkers. **(a)** Relative mRNA expression of BNP in cardiac tissue determined by RT-qPCR, **(b)** serum CK-MB activity, **(c)** serum MDA levels, and **(d)** SOD activity. Values are expressed as mean ± SEM (n = 8). *p < 0.05 vs. control group; ^#^*p* < 0.05 vs. DCM group; ^$^*p* < 0.05 vs. Losartan-treated group. BNP: brain natriuretic peptide; CK-MB: creatine kinase-MB; MDA: malondialdehyde; SOD: superoxide dismutase.

Treatment with OL and Losartan significantly reduced BNP expression and CK-MB activity relative to untreated diabetic rats, suggesting attenuation of myocardial injury and improvement of cardiac cellular integrity (Fig. 5a, b).

Diabetic rats also demonstrated pronounced oxidative stress, as evidenced by significantly increased serum MDA levels and reduced SOD activity compared to control animals. Both OL and Losartan significantly ameliorated these alterations by decreasing MDA levels and restoring SOD activity. Interestingly, OL treatment resulted in a greater reduction in MDA levels compared to Losartan, suggesting stronger antioxidant activity (Fig. 5c, d).

### Cardiac remodeling, fibrosis and TGF-β1/Smad signaling

Gene expression analysis demonstrated significant upregulation of multiple fibrosis-related markers and downstream markers in the myocardium of diabetic rats. The DCM group exhibited increased expression of α-SMA, collagen-I, collagen-III, MMP-2, MMP-9, and TIMP-1 compared to control animals, indicating enhanced ECM remodeling and fibrotic progression.

**Fig. 6:**
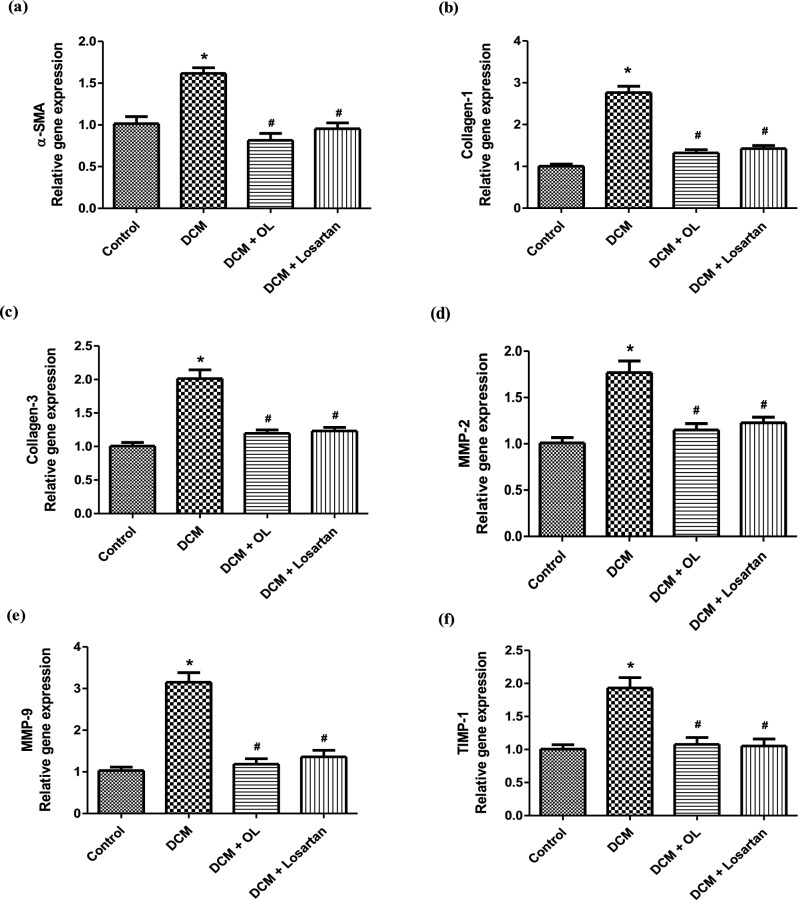
Expression of fibrosis-related genes in cardiac tissue. Relative mRNA expression levels of fibrosis-related genes determined by RT-qPCR: **(a)** α-SMA, **(b)** collagen-I, **(c)** collagen-III, **(d)** MMP-2, **(e)** MMP-9, and **(f)** TIMP-1. Gene expression levels were normalized to the housekeeping gene and expressed relative to the control group. Values are expressed as mean ± SEM (n = 8). *p < 0.05 vs. control group; ^#^*p* < 0.05 vs. DCM group. α-SMA: alpha-smooth muscle actin; MMP: matrix metalloproteinase; TIMP: tissue inhibitor of metalloproteinase.

Treatment with OL and Losartan significantly downregulated the expression of these profibrotic genes relative to the untreated DCM group, suggesting attenuation of myocardial fibrosis and cardiac remodeling (Fig. 6a–f).

**Fig. 7:**
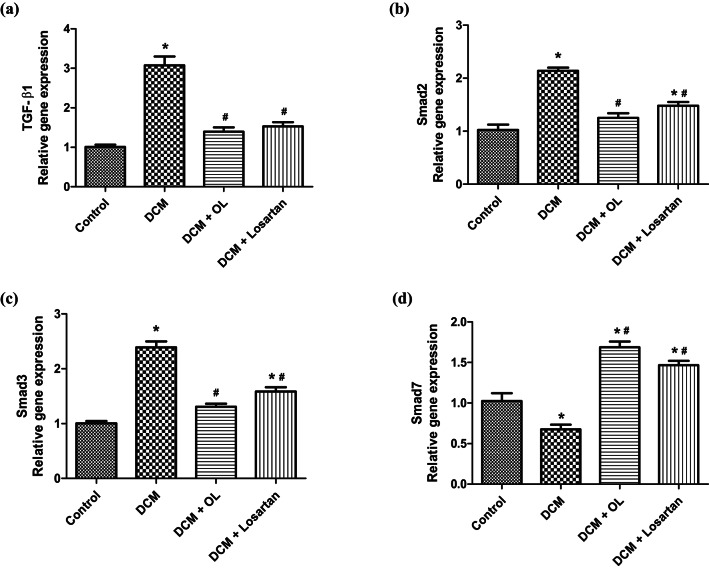
Modulation of TGF-β1/Smad signaling pathway in cardiac tissue. Relative mRNA expression levels of **(a)** TGF-β1, **(b)** Smad2, **(c)** Smad3, and **(d)** Smad7 determined by RT-qPCR. Gene expression levels were normalized to the housekeeping gene and expressed relative to the control group. Values are expressed as mean ± SEM (n = 8). *p < 0.05 vs. control group; ^#^*p* < 0.05 vs. DCM group. TGF-β1: transforming growth factor beta-1; *p*-Smad3: phosphorylated Smad3; GAPDH: Glyceraldehyde 3-phosphate dehydrogenase.

Further mechanistic investigation revealed activation of the TGF-β1/Smad signaling pathway in diabetic myocardium. Diabetic rats exhibited significant upregulation of TGF-β1, Smad2, and Smad3 gene expression accompanied by reduced expression of the inhibitory mediator Smad7 (Fig. 7a–d). These transcriptional changes were supported by protein-level analyses. TGF-β1 protein expression increased significantly in the DCM group relative to controls (Mean Difference =  + 5.40 fold, 95% CI [4.37 to 6.43], *p* < 0.001), while treatment with OL and Losartan significantly reduced its expression (OL vs. DCM: Mean Difference = − 4.17 fold, 95% CI [− 5.20 to − 3.14], *p* < 0.001; Losartan vs. DCM: Mean Difference =  – 3.45 fold, 95% CI [− 4.48 to − 2.42], *p* < 0.001). Similarly, *p*-Smad3 protein levels were significantly elevated in diabetic rats compared to controls (Mean Difference =  + 7.05 fold, 95% CI [5.82 to 8.27], *p* < 0.001), and both treatments markedly attenuated Smad3 phosphorylation (OL vs. DCM: Mean Difference = − 5.08 fold, 95% CI [− 6.30 to − 3.85], *p* < 0.001; Losartan vs. DCM: Mean Difference = − 4.09 fold, 95% CI [− 5.32 to − 2.87], *p* < 0.001) (Fig. S2).

Together, these findings indicate that diabetes-induced activation of the TGF-β1/Smad signaling pathway contributes to myocardial fibrosis and that OL and Losartan exert cardioprotective effects, at least in part, through suppression of this profibrotic signaling cascade.

## Discussion

The present study demonstrates that treatment with OL ameliorates multiple pathological features of experimental DCM. OL administration was associated with improvements in metabolic control, attenuation of oxidative stress, preservation of cardiac structure, and a significant reduction in myocardial fibrosis. Functional improvement was reflected by normalization of echocardiographic parameters, including reductions in LV dilation and improved EF%. Histological and molecular analyses further demonstrated decreased collagen deposition, reduced FN accumulation, and suppression of profibrotic gene expression in cardiac tissue. Collectively, these findings suggest that OL improves ventricular compliance and contractility primarily through attenuation of cardiac fibrosis. In addition, our results provide insight into a potential therapeutic mechanism, demonstrating that OL suppresses TGF-β1/Smad signaling in cardiac tissue. While this pathway appears central to the antifibrotic action of OL, additional mechanisms, including modulation of oxidative stress and systemic metabolic regulation, likely contribute to its overall cardioprotective profile.

Metabolic dysregulation is a major contributor to the development of DCM. In the present study, diabetic animals exhibited hyperglycemia, impaired insulin secretion, and increased HOMA-IR, reflecting insulin resistance accompanied by partial β-cell dysfunction. OL treatment significantly improved glycemic parameters and partially restored circulating insulin levels (Table 1). These metabolic improvements likely contributed to the cardioprotective profile observed in treated animals. Previous studies suggest that OL’s antidiabetic effects may be attributed to its antioxidant activity; improving insulin secretion possibly through protection of pancreatic β-cells against STZ-induced oxidative damage^31,32^. Additionally, OL has been reported to reduce HFD-induced insulin resistance and stimulate glucose transporter type 4 (GLUT4) translocation to skeletal muscle membranes, thereby enhancing peripheral glucose uptake^33^. The improved glucose metabolism may also explain the partial protection against weight loss observed in diabetic rats, as improved metabolic status may reduce protein and lipid catabolism associated with insulin deficiency or resistance^34^. Similar antihyperglycemic effects of OL have been reported in other experimental models of metabolic disorders, further supporting its role in metabolic regulation^31,33,35^.

Oxidative stress is a major contributor to myocardial injury in diabetes, promoting cellular damage, mitochondrial dysfunction, and activation of profibrotic signaling pathways. In the present study, diabetic rats exhibited increased lipid peroxidation, reflected by elevated MDA levels, together with reduced activity of the antioxidant enzyme SOD. OL treatment markedly reduced oxidative stress markers and restored antioxidant capacity (Fig. 5). These findings are consistent with the well-established antioxidant properties of OL, which arise from its phenolic catechol structure capable of scavenging reactive oxygen species and stabilizing free radicals^36^. Previous studies have similarly demonstrated that OL reduces oxidative stress in various models of cardiac injury, including 5-fluorouracil-, cisplatin-, and ketamine-induced cardiotoxicity^37–39^, where it decreased MDA levels, restored endogenous antioxidant enzymes, and improved myocardial histology. Collectively, these findings suggest that attenuation of oxidative stress represents an important mechanism underlying OL-mediated cardioprotection.

Beyond systemic metabolic and antioxidant effects, OL exerted significant structural benefits within the myocardium. Diabetic animals developed characteristic features of cardiac remodeling, including LV dilation, increased HW/BW ratio, elevated BNP levels, and extensive interstitial fibrosis. These alterations were substantially attenuated following OL treatment. Histological analysis demonstrated improved myocardial architecture and reduced collagen accumulation (Figs. 2, 3), while immunohistochemical staining revealed decreased FN and α-SMA expression (Fig. 4), indicating reduced myofibroblast activation. Consistent with these observations, OL significantly downregulated key profibrotic genes including collagen I, collagen III, MMP-2, MMP-9, and TIMP-1 (Fig. 6). These findings support the hypothesis that OL attenuates ECM remodeling and fibrotic progression in the diabetic myocardium.

The antifibrotic effects observed in this study may be partially mediated through modulation of the TGF-β1/Smad signaling pathway, a central regulator of cardiac fibrosis. Diabetic rats exhibited marked activation of this pathway, characterized by increased expression of TGF-β1 and its downstream mediators Smad2 and Smad3 together with suppression of the inhibitory Smad7. OL treatment significantly reduced TGF-β1 expression and Smad3 phosphorylation while restoring Smad7 levels (Fig. 7). These molecular changes were accompanied by reduced expression of fibrosis-associated genes and decreased collagen deposition, suggesting a functional association between pathway modulation and attenuation of myocardial fibrosis. Nevertheless, it is possible that the observed modulation of TGF-β1/Smad signaling occurs secondary to improvements in oxidative stress and metabolic status rather than through direct molecular inhibition by OL.

Previous studies further support the ability of OL to modulate signaling pathways involved in cardiac remodeling and injury. Experimental investigations have shown that OL activates intracellular cardioprotective pathways such as PI3K/Akt, AMPK, and endothelial nitric oxide synthase signaling, which contribute to improved myocardial energy metabolism and reduced oxidative stress^40^. OL has also been reported to ameliorate endoplasmic reticulum stress through sirtuin-1 activation, providing protection against cardiac hypertrophy and apoptosis^41^, and to suppress inflammatory mediators including TNF-α and IL-6 in models of cardiotoxicity and systemic inflammation^37,38^. In addition, emerging evidence suggests that OL may interfere with collagen fibril formation and ECM assembly in vitro^42^, providing a possible direct mechanism for its antifibrotic properties. OL has also been shown to suppress ECM-related gene expression in endothelial and fibroblast cells^43–45^. Together, these findings support the concept that OL exerts cardioprotective effects through multiple complementary mechanisms involving antioxidant, metabolic, anti-inflammatory, and antifibrotic actions.

In the present study, Losartan was included as a positive control reference treatment due to its well-established antifibrotic and cardioprotective effects mediated through inhibition of the renin-angiotensin system and modulation of TGF-β signaling^46–48^. Both OL and Losartan attenuated cardiac fibrosis and improved functional parameters. However, due to differences in pharmacological mechanisms and dosing regimens, direct conclusions regarding therapeutic superiority cannot be drawn. Rather, the comparison provides pharmacological context for interpreting the magnitude of OL-induced cardioprotective effects within the experimental conditions of the present study.

Overall, the present findings contribute to the growing body of evidence supporting the cardioprotective potential of natural polyphenols in diabetic heart disease. Bioactive compounds such as Resveratrol^12,49^, Curcumin^13,50^, and Embelin^51^ have previously demonstrated antioxidant and antifibrotic properties in experimental models of DCM. Within this broader therapeutic landscape, the antifibrotic efficacy of OL is highly competitive, displaying a comprehensive modulation of the TGF-β1/Smad cascade that is comparable to these established agents. OL appears particularly promising due to its dual ability to improve systemic metabolic and oxidative status while simultaneously attenuating myocardial fibrosis.

While the present *in vivo* study demonstrates significant cardioprotective and antifibrotic effects of OL, the relationship between OL administration and TGF-β1/Smad signaling modulation must be interpreted as an association rather than demonstrated direct causality. Because OL significantly improved systemic blood glucose profiles and reduced global oxidative stress, it is probable that the observed attenuation of the TGF-β1/Smad pathway is, at least in part, secondary to the amelioration of the diabetic microenvironment rather than exclusive direct inhibition of the pathway itself. However, the pronounced reduction in specific ECM proteins (collagen and FN) relative to the more moderate glycemic improvements suggests OL exerts overlapping, direct myocardial benefits. This is further supported by previously reported *in vitro* studies demonstrating the suppression of ECM-related gene expression by OL in isolated fibroblasts^39–41^, strongly suggesting an independent, direct antifibrotic effect within the myocardium.

Beyond mechanistic considerations, several critical translational limitations must be acknowledged. The relevance of this animal model to human DCM is constrained by inherent species differences, the reliance on a single-sex cohort, a relatively short observation period that does not mimic decades of human disease progression, and the absence of complex clinical comorbidities typically seen in human DCM. Regarding therapeutic translatability, body surface area normalization of the 40 mg/kg animal dose yields a Human Equivalent Dose (HED) of approximately 6.48 mg/kg for an adult, which is a pharmacologically achievable target. Nevertheless, comprehensive preclinical validation, including investigations into dose-response relationships, long-term administration, alternative oral routes, and the inclusion of both sexes, will be necessary to fully establish the therapeutic efficacy of OL prior to clinical translation.

## Conclusion

This study demonstrates that therapeutic administration of OL exerts significant cardioprotective effects in experimental DCM by improving systemic metabolic control, reducing oxidative stress, and profoundly attenuating myocardial fibrosis. These antifibrotic effects are strongly associated with the suppression of the TGF-β1/Smad signaling cascade, resulting in reduced ECM deposition and the significant preservation of LV structure and function. However, while these findings highlight OL as a promising natural therapeutic candidate, its modulation of the TGF-β1/Smad pathway must currently be interpreted as a robust association rather than demonstrated direct causality. To advance OL toward clinical applicability, future research should prioritize direct mechanistic validation, evaluate long-term efficacy in models encompassing diverse human comorbidities, and investigate its potential as an adjunct to established pharmacological therapies.

## Supplementary Information

Supplementary Information.

## Data Availability

The datasets of the current study are available from the corresponding author on request.

## Abbreviations

α-SMA: α-Smooth muscle actin
ANOVA: One-way analysis of variance
AT1R: Angiotensin II type 1 receptor
BNP: B-type natriuretic peptide
BSA: Bovine serum albumin
BW: Body weight
CI: Confidence interval
CK-MB: Creatine kinase-MB
Ct: Cycle threshold
DAB: 3, 3′-Diaminobenzidine
DCM: Diabetic cardiomyopathy
ECL: Enhanced chemiluminescence
ECM: Extracellular matrix
EF%: Ejection fraction
FN: Fibronectin
GAPDH: Glyceraldehyde 3-phosphate dehydrogenase
GLUT4: Glucose transporter type 4
H&E: Hematoxylin and eosin
HED: Human Equivalent Dose
HFD: High-fat diet
HOMA-IR: Insulin resistance index
HW: Heart weight
i.p.: Intraperitoneal
LV: Left ventricular
LVDd: Left ventricular diameter at end-diastole
LVDs: Left ventricular diameter at end-systole
MDA: Malondialdehyde
MMP: Matrix metalloproteinases
NODCAR: National Organization for Drug Control and Research
OL: Oleuropein
PBS: Phosphate-buffered saline
p-SMAD3: Phosphorylated SMAD3
PVDF: Polyvinylidene fluoride
SDS-PAGE: SDS–polyacrylamide gel electrophoresis
SEM: Standard error of the mean
SOD: Superoxide dismutase
STZ: Streptozotocin
TBST: Tris-buffered saline containing 0.1% Tween-20
T2DM: Type 2 diabetes milieus
TGF-β1: Transforming growth factor beta-1
TIMP-1: Tissue inhibitor of metalloproteinases-1
