# Supplementary material for: Oleuropein attenuates cardiac fibrosis via modulation of TGF-β1/Smad pathway in diabetic cardiomyopathy rat model
Source: Sci Rep. 2026 May 18;16:15350. doi: 10.1038/s41598-026-49571-3 (PMC13184247; doi:10.1038/s41598-026-49571-3)

Table S1. Composition of chow and high-fat diet (HFD)

Standard chow diet			
**Macronutrient**	**Amount (g/100g)**	**Energy (kcal/100g)**	**% of Total Calories**
Carbohydrates	50	200	66.67%
Proteins	16	64	21.33%
Fats	4	36	12.00%
**Total**		**3 kcal/g**	
HFD preparation (70 g standard chow mixing with 28 g sheep-tail fat)			
**Macronutrient**	**Amount (g/70g)**	**Energy (kcal/g)**	**% of Total Calories**
Carbohydrates	35 g	140	~30.3%
Proteins	11.2 g	44.8	~9.7%
Fats	2.8 g<br>**+ 28 g Sheep-tail (almost pure fat)**	277.2	~60%
**Total**		**4.62 kcal/g**	

Table S2. Absolute Mean Differences and 95% Confidence Intervals for remaining systemic, gene, and protein expression

**Parameter**	**Control vs. DCM (Disease Induction)**<br>**(Mean Diff [95% CI], p-value)**	**DCM vs. OL (Treatment Effect)**<br>**(Mean Diff [95% CI], p-value)**	**DCM vs. Losartan (Treatment Effect)**<br>**(Mean Diff [95% CI], p-value)**
**Metabolic Markers**			
Fasting Insulin (ng/ml)	-0.61 [-0.98 to -0.23], p<0.001	+0.39 [+0.01 to +0.77], p<0.05	+0.36 [-0.02 to +0.73], ns
HOMA-IR	+9.28 [+6.73 to +11.82], p<0.001	-6.95 [-9.49 to -4.41], p<0.001	-6.38 [-8.92 to -3.83], p<0.001
Body Weight (g)	-111.1 [-173.8 to -48.49], p < 0.001	-15.38 [-78.01 to 47.26], ns	-23.00 [-85.63 to 39.63], ns
**Echocardiography**			
LVDd (mm)	+2.16 [1.02 to 3.31], p < 0.001	-1.66 [-2.81 to -0.52], p < 0.01	-1.35 [-2.49 to -0.21], p < 0.05
LVDs (mm)	+1.89 [1.19 to 2.58], p < 0.001	-1.69 [-2.38 to -0.99], p < 0.001	-1.46 [-2.16 to -0.77], p < 0.001
**Injury & Oxidative Stress**			
Serum CK-MB (U/L)	+162.9 [124.3 to 201.5], p<0.001	-136.8 [-175.4 to -98.17], p<0.001	-120.5 [-159.1 to -81.90], p<0.001
Serum MDA (nmol/ml)	+82.50 [64.97 to 100.0], p<0.001	-66.80 [-84.33 to -49.27], p<0.001	-53.88 [-71.41 to -36.34], p<0.001
Serum SOD (U/ml)	-16.06 [-21.29 to -10.83], p<0.001	+11.24 [+6.01 to +16.46], p<0.001	+9.12 [+3.90 to +14.35], p<0.001
BNP mRNA (Fold Change)	+1.20 [0.89 to 1.51], p<0.001	-0.90 [-1.22 to -0.59], p<0.001	-0.72 [-1.04 to -0.41], p<0.001

**Profibrotic Gene Expression (RT-qPCR)**			
α-SMA mRNA (Fold Change)	+0.60 [0.30 to 0.90], p<0.001	-0.80 [-1.10 to -0.50], p<0.001	-0.66 [-0.96 to -0.36], p<0.001
Collagen I mRNA (Fold Change)	+1.76 [1.39 to 2.14], p<0.001	-1.44 [-1.82 to -1.07], p<0.001	-1.34 [-1.71 to -0.97], p<0.001
Collagen III mRNA (Fold Change)	+1.01 [0.69 to 1.32], p<0.001	-0.82 [-1.14 to -0.50], p<0.001	-0.78 [-1.10 to -0.47], p<0.001
MMP-2 mRNA (Fold Change)	+0.76 [0.43 to 1.09], p < 0.001	-0.62 [-0.95 to -0.29], p < 0.001	-0.54 [-0.87 to -0.21], p < 0.001
MMP-9 mRNA (Fold Change)	+2.12 [1.45 to 2.78], p<0.001	-1.97 [-2.63 to -1.30], p<0.001	-1.79 [-2.45 to -1.12], p<0.001
TIMP-1 mRNA (Fold Change)	+0.93 [0.45 to 1.40], p<0.001	-0.86 [-1.33 to -0.38], p<0.001	-0.88 [-1.35 to -0.41], p<0.001
**TGF-β1/Smad Pathway (RT-qPCR)**			
TGF-β1 mRNA (Fold Change)	+2.07 [1.52 to 2.61], p<0.001	-1.68 [-2.22 to -1.13], p<0.001	-1.55 [-2.09 to -1.00], p<0.001
Smad2 mRNA (Fold Change)	+1.12 [0.79 to 1.44], p<0.001	-0.89 [-1.21 to -0.56], p<0.001	-0.66 [-0.98 to -0.33], p<0.001
Smad3 mRNA (Fold Change)	+1.38 [1.08 to 1.69], p<0.001	-1.08 [-1.39 to -0.78], p<0.001	-0.80 [-1.11 to -0.50], p<0.001
Smad7 mRNA (Fold Change)	-0.35 [-0.63 to -0.06], p<0.05	+1.01 [+0.73 to +1.29], p<0.001	+0.79 [+0.51 to +1.07], p<0.001
**Immunohistochemistry (IHC)**			
Fibronectin (Area %)	+61.70 [+44.94 to +78.46], p<0.001	-55.39 [-72.15 to -38.63], p<0.001	-33.56 [-50.32 to -16.80], p<0.01
α-SMA (Area %)	+28.19 [+22.93 to +33.45], p<0.001	-24.04 [-29.30 to -18.78], p<0.001	-20.16 [-25.42 to -14.90], p<0.001

Fig. S1

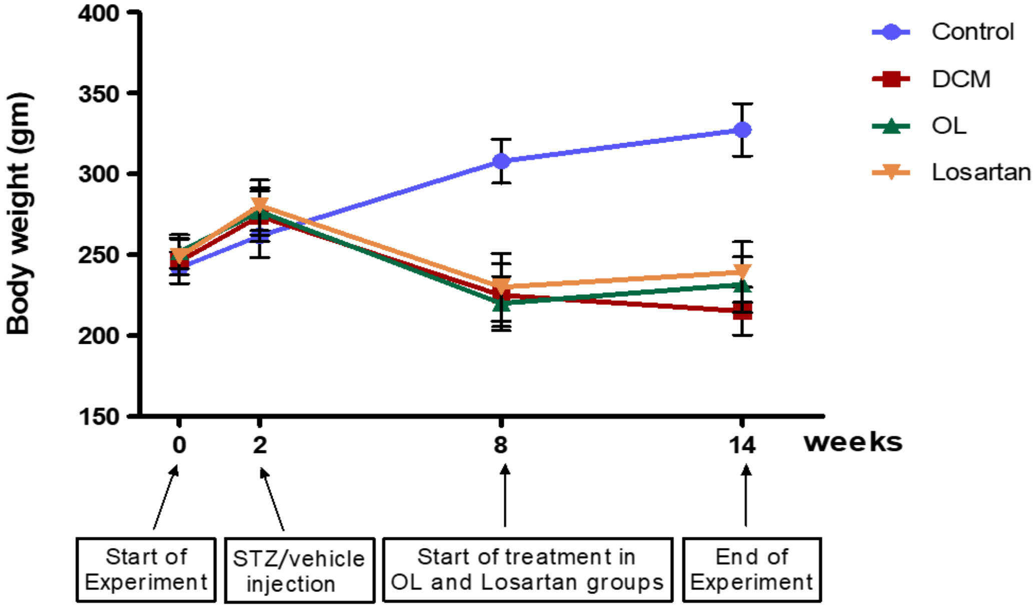

Fig. S1: Body weight changes during the experimental period

During the first 2 weeks, all animals demonstrated a trend of increasing body weight. Following STZ injection at the end of week 2, a decline was observed in the body weight of the 3 diabetic groups receiving HFD/STZ (non-treated DCM, OL-treated, and Losartan-treated). By the end of the study, OL and Losartan treatments produced a modest tendency toward weight gain; however, this effect did not reach statistical significance compared with the diabetic non-treated (DCM) group

Fig. S2

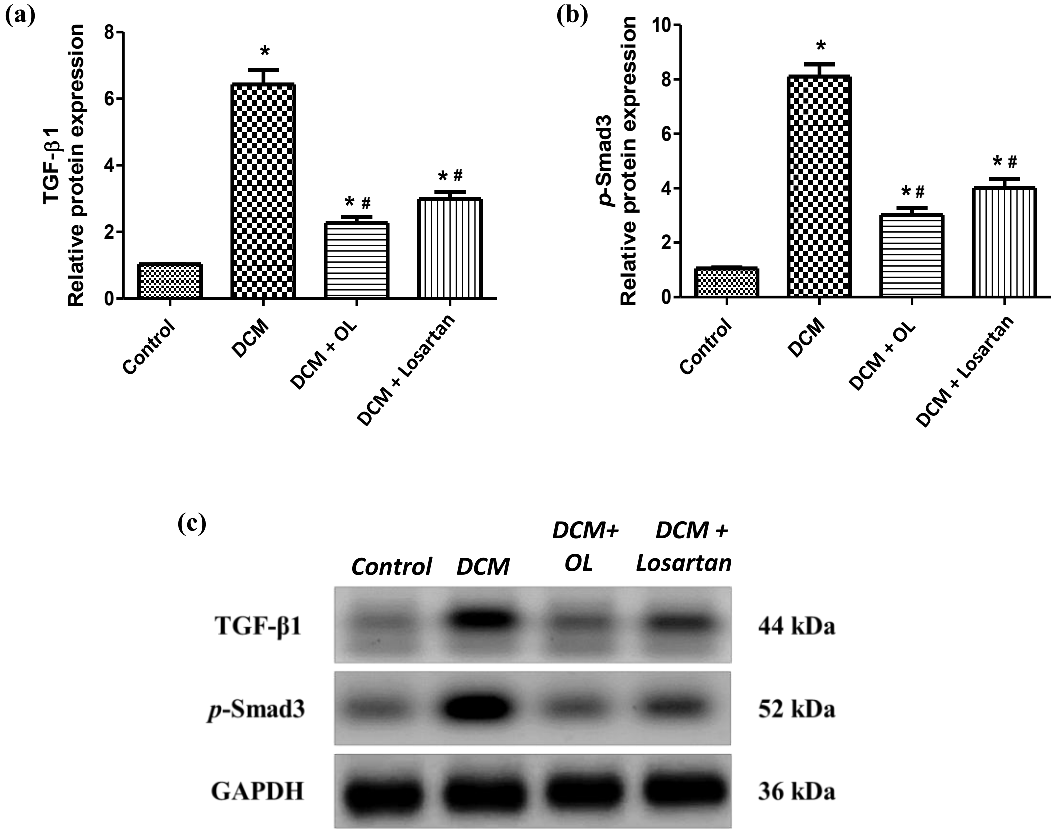

Fig S2. Modulation of TGF-β1/Smad signaling pathway in cardiac tissue

Quantitative protein expression analysis of **(a)** TGF-β1 and **(b)** *p*-Smad3 determined by western blot densitometry. **(c)** Representative western blot bands corresponding to the analyzed proteins. Target proteins and their respective loading controls (GAPDH) were processed from parallel gels run under identical experimental conditions using the same protein lysates. Horizontal white space indicates that proteins were detected on separate gels. Values are expressed as mean ± SEM (n=8). *$p < 0.05$ vs. control group; $^{\#}p < 0.05$ vs. DCM group.

TGF-β1: transforming growth factor beta-1; p-Smad3: phosphorylated Smad3; GAPDH: Glyceraldehyde 3-phosphate dehydrogenase.

Western blot original available blot images and densitometry data:

Sample	Target TGF-b	Average	loading control	Average	Normalization to loading control	Normalization to experimental control
C1	2726.8	3735.0333	22405.3	20414.5	0.182959824	1.025130104
C1	4462.9		20412.5			
C1	4015.4		18425.7			
C2	3498.2	3872.1	23895.8	21897.0667	0.176831904	0.990795161
C2	3797.6		21945.2			
C2	4320.5		19850.2			
C3	3098.7	4011.2667	20920.6	22977.2333	0.174575703	0.978153589
C3	4150.5		23025.4			
C3	4784.6		24985.7			
C4	3164.7	3113.7	21120.8	21078.9333	0.147716203	0.827658899
C4	3624.6		19020.4			
C4	2551.8		23095.6			
C5	3724.9	3877.2333	22410.3	22373.8667	0.173292949	0.970966273
C5	4085.2		20325.9			
C5	3821.6		24385.4			
C6	3540.8	3932.2667	17850.8	19885.8	0.197742443	1.107957616
C6	3774.8		21885.9			
C6	4481.2		19920.7			
C7	4112.4	4329.9667	23640.2	23583.7	0.183599972	1.028716875
C7	4951.6		21515.6			
C7	3925.9		25595.3			
C8	4631.5	3936.3333	17205.2	20600.5667	0.191078886	1.070621483
C8	3894.8		21310.7			
C8	3282.7		23285.8			
				Average=	**0.178474736**	**1**

Sample	Target TGF-b	Average	loading control	Average	Normalization to loading control	Normalization to experimental control
D1	24120.8	26047.4	20785.4	17912.7	1.45413031	8.147541468
D1	25036		16025.6			
D1	28985.4		16927.1			
D2	26550.7	28478.5	16350.2	18280.2	1.557887769	8.728898035
D2	27464.2		17279.8			
D2	31420.6		21210.6			
D3	19784.8	20787	18163.2	19161.5333	1.084829676	6.078337487
D3	18850.3		22120.9			
D3	23725.9		17200.5			
D4	26750.1	23786.633	21453.8	22461.6333	1.058989477	5.933553973
D4	22789.3		25410.7			

D4	21820.5		20520.4			
D5	24687.9	25686.333	28620.5	25618.5667	1.002645217	5.617855173
D5	28620.7		23600.8			
D5	23750.4		24634.4			
D6	19600.2	16659.733	18200.6	20141.5333	0.82713332	4.634456058
D6	14720.6		23100.2			
D6	15658.4		19123.8			
D7	20483	21484.9	22343.9	23365	0.91953349	5.152177348
D7	24450.8		21400.7			
D7	19520.9		26350.4			
D8	18800.5	21074.4	19895.6	16946.9	1.243554868	6.967680125
D8	23695.6		15924.2			
D8	20727.1		15020.9			
					Average=	**6.407562458**

Sample	Target TGF-b	Average	loading control	Average	Normalization to loading control	Normalization to experimental control
OL1	3105.7	5001.4333	18320.7	20189.5667	0.247723659	1.388004069
OL1	4003.4		22985.6			
OL1	7895.2		19262.4			
OL2	12850.6	13123.933	24567.4	25563.0333	0.513394994	2.87656957
OL2	17620.9		28520.9			
OL2	8900.3		23600.8			
OL3	6900.8	8698.7667	24725.4	21119.4333	0.411884473	2.307802678
OL3	11520.4		18850.2			
OL3	7675.1		19782.7			
OL4	10839.5	11337.467	27185.3	24204.4667	0.468403903	2.624482965
OL4	9590.2		22200.6			
OL4	13582.7		23227.5			
OL5	6205.9	8105.2667	20395.7	17449.9333	0.46448697	2.602536256
OL5	10985.6		15500.9			
OL5	7124.3		16453.2			
OL6	5098.8	7690.6333	19620.5	22218.8333	0.346131285	1.939385335
OL6	6152.4		20550.2			
OL6	11820.7		26485.8			
OL7	7250.2	8584.8333	23495.6	20683.5333	0.415056422	2.325575215
OL7	10195.5		18600.3			
OL7	8308.8		19954.7			
OL8	11420.3	8332.5667	17920.7	21533.2	0.386963696	2.168170721
OL8	6069.6		20858.4			
OL8	7507.8		25820.5			
					Average=	**2.279065851**

Sample	Target TGF-b	Average	loading control	Average	Normalization to loading control	Normalization to experimental control
L1	17895.6	15233.433	22628.7	23618.2333	0.644986148	3.613879272
L1	14384		21705.4			
L1	13420.7		26520.6			
L2	12752.8	15391.233	27985.7	25377.6	0.606488925	3.398178024
L2	19220.4		23100.8			
L2	14200.5		25046.3			
L3	15485.7	12269.733	18600.9	20524.4667	0.597810093	3.349550238
L3	11922.6		23450.2			
L3	9400.9		19522.3			
L4	10497.8	11361.133	25285.4	22340.9333	0.508534409	2.849335551
L4	13985.2		20400.5			
L4	9600.4		21336.9			
L5	13200.5	10312.467	23120.6	25049.5667	0.411682438	2.306670667
L5	8420.7		24032.8			
L5	9316.2		27995.3			
L6	10266.6	13095.433	22510.9	23532.4667	0.556483667	3.117996867
L6	12924.4		21600.7			
L6	16095.3		26485.8			
L7	11620.9	9872.2333	19959.1	20966.7	0.470852988	2.638205266
L7	9050.8		23920.6			
L7	8945		19020.4			
L8	6500.9	8775	21985.7	19003.9	0.461747326	2.587185933
L8	11405.7		17975.8			
L8	8418.4		17050.2			
					Average=	**2.982625227**

Sample	Target p-smad3	Average	loading control	Average	Normalization to loading control	Normalization to experimental control
C1	4464.5	4020.5	19606.8	21396.7	0.187902807	1.198568422
C1	4230.1		21812.2			
C1	3366.8		22771.1			
C2	4475.9	3571.2	14682	17070.3	0.209205462	1.334450847
C2	4474.5		17351.3			
C2	1763.2		19177.6			
C3	1029.5	2955.8	23081.7	24513.5	0.120578457	0.769129172
C3	4460.3		24544.5			
C3	3377.6		25914.3			
C4	1013.9	3278.4	16865.8	19250.2	0.170304724	1.086316204
C4	4359.5		18684.2			
C4	4461.7		22200.6			

C5	2435.1	2100.7	21541.8	23110.9	0.090896503	0.579798036
C5	2717.8		23051.7			
C5	1149.2		24739.2			
C6	2990.7	3083.9	21709.7	20950.2	0.147201459	0.938948292
C6	2329.1		22570.9			
C6	3931.8		25052.1			
C7	3853.1	4105.3	24137	26501.4	0.154908797	0.988110796
C7	4117.3		26537.1			
C7	4345.5		28830.1			
C8	3805.1	3894.6	20021.4	22488.3	0.173183389	1.104678231
C8	3397.4		22767.3			
C8	4481.3		24676.2			
				Average=	**0.1567727**	**1**

Sample	Target p-smad3	Average	loading control	Average	Normalization to loading control	Normalization to experimental control
D1	27681.8	26845.2	13659.5	17411.3	1.541826285	9.834788117
D1	30072.6		18244.6			
D1	22781.2		20329.8			
D2	22446.6	25709.4	18846.2	22300.3	1.152872383	7.353782797
D2	29438.1		22927			
D2	25243.5		25127.7			
D3	33264.3	29661.9	15499.4	18246.7	1.625603534	10.36917484
D3	28932.2		18263.6			
D3	26789.2		20977.1			
D4	20016.6	23800.5	15055	17334.7	1.372997514	8.757886515
D4	24169.5		17733.4			
D4	27215.4		19215.7			
D5	24225.5	24778	21674	24622.9	1.006299014	6.41884088
D5	22838.9		25495.9			
D5	27269.6		26698.8			
D6	17939.2	19627.4	15873.1	19178.2	1.023422428	6.528065338
D6	19182.3		19220.4			
D6	21760.7		22441.1			
D7	20762.7	20885.3	19306.8	22351.8	0.934390071	5.960158067
D7	18662.6		22958.2			
D7	23230.6		24790.4			
D8	27029.8	23950.1	13215.4	15950.5	1.501526598	9.577730049
D8	23823.9		16422.6			
D8	20996.6		18213.5			
					Average=	**8.100053325**

Sample	Target p-smad	Average	loading control	Average	Normalization to loading control	Normalization to experimental control
OL1	5178.5	6775.1	16588.2	19355.1	0.350042108	2.232800151
OL1	6686.7		19131			
OL1	8460.1		22346.1			
OL2	14310.9	13850.3	18705.5	21045.9	0.658099677	4.19779514
OL2	15645.8		20904.8			
OL2	11594.2		23527.4			
OL3	6166.9	7709.5	18507.2	20974.7	0.367561872	2.344552799
OL3	9407.8		21264.8			
OL3	7553.8		23152.1			
OL4	12894.4	10850.1	19881.7	23280.3	0.466063582	2.972861871
OL4	8941.8		23668.6			
OL4	10714.1		26290.6			
OL5	7326	9133.4	15193.3	16551.9	0.55180372	3.519769201
OL5	8613.4		16245			
OL5	11460.8		18217.4			
OL6	12959.6	12942.7	17778.3	20500.7	0.631329662	4.027038271
OL6	15172.1		19788.6			
OL6	10696.4		23935.2			
OL7	6625.5	8720	20205.9	22307.1	0.390906931	2.493463029
OL7	10833.6		22760.2			
OL7	8700.9		23955.2			
OL8	9843.3	9511.5	22413.1	25338.5	0.37537739	2.394405343
OL8	11889.8		24916.4			
OL8	6801.4		28686			
					Average=	**3.022835726**

Sample	Target p-smad3	Average	loading control	Average	Normalization to loading control	Normalization to experimental control
L1	14524.1	14478.2	12254.3	16032.1	0.903075704	5.760414316
L1	17491.9		16159.6			
L1	11418.6		19682.4			
L2	15563.8	15754.3	17679.3	21050.9	0.748390805	4.77373169
L2	13084.6		21241.2			
L2	18614.5		24232.2			
L3	8379.9	11920.5	17470.7	19522.3	0.610609406	3.894870768
L3	12050.3		19511.4			
L3	15331.3		21584.8			
L4	10290.9	10551.8	17221.6	20354.1	0.518411524	3.30677168
L4	13274.3		20663.9			

L4	8090.2		23176.8			
L5	5960.1	9953.6	18964.1	22039.4	0.45162754	2.880779245
L5	10597.8		21839.3			
L5	13303		25314.8			
L6	17334.9	13920.1	19776.8	22141.2	0.628696728	4.010243678
L6	11419.3		22265.8			
L6	17334.9		24381			
L7	14495.3	14322.8	15794.7	19265	0.743462237	4.742294023
L7	13006.1		19628.4			
L7	17262.5		22371.9			
L8	5154.4	7425.1	15448.3	17811.8	0.416864101	2.659035035
L8	9389.2		17915.7			
L8	7731.7		20071.4			
					Average=	**4.003517554**

Blot for TGF-β

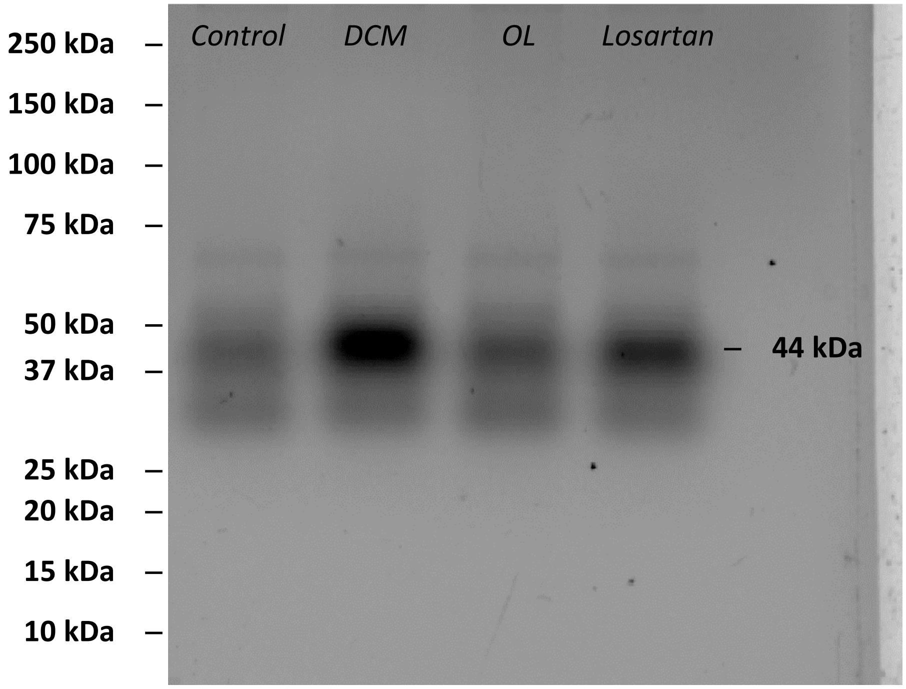

Blot for *p*-SMAD3

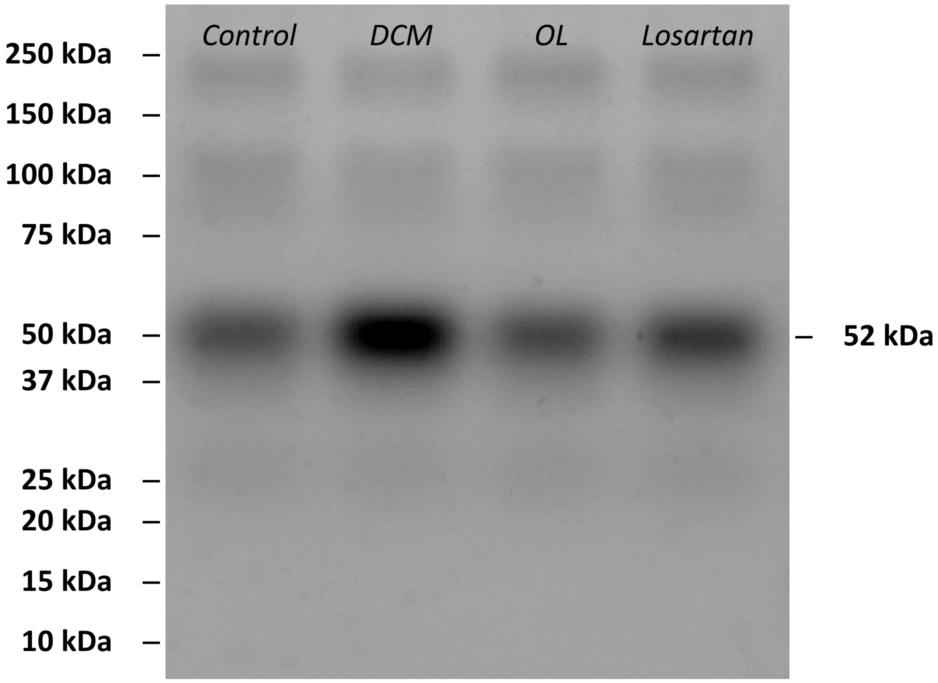

Blot for control (GADPH)

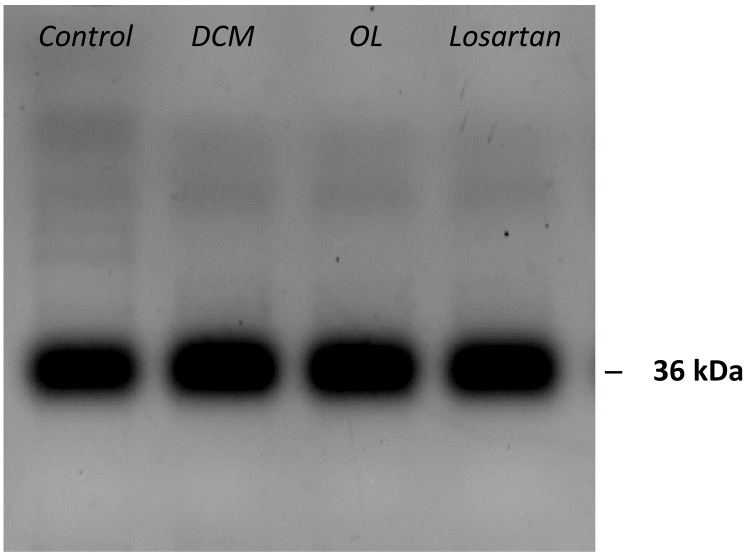

Supplement: Supplementary file 1 — Supplementary Information. [file 41598_2026_49571_MOESM1_ESM.pdf]
